# Reviewed article: Mota DM, Leitão LO, Silva RS, Fernandes CFR, Silva MT. Actions to mitigate ocular adverse events related to hair styling cosmetics in Brazil: a descriptive and correlational study (2022-2024). Epidemiol Serv Saude.

**DOI:** 10.1590/S2237-96222025v34e20240449.a

**Published:** 2025-05-02

**Authors:** Thais Machado

**Affiliations:** FOUSP, Pediatric Dentistry

**Table:** 

Rating	Excellent	Good	Average	Below average	Poor
Interest		✓			
Quality	✓				
Originality		✓			
Overall	✓				

**Table:** 

Questionnaire	Yes	No	Not applicable
Does the manuscript contain new and significant information to justify publication?	✓		
Does the Abstract (Summary) clearly and accurately describe the content of the article?	✓		
Is the problem significant and concisely stated?		✓	
Are the methods described comprehensively?	✓		
Are the interpretations and conclusions justified by the results?	✓		
Is adequate reference made to other work in the field?		✓	

## Manuscript Structure:

Length of article is: Adequate

Number of tables is: Too short

Number of figures is: Adequate

Please state any conflict(s) of interest that you have in relation to the review of this paper (state “none” if this is not applicable): None

## Comments

Prezados Autores,

Agradecemos pelo envio do seu artigo para revisão. Após análise, destacamos os seguintes pontos que precisam ser abordados antes da publicação:

O texto é original, com uma similaridade inferior a 30% no iThenticate. O uso de inteligência artificial foi adequadamente informado na seção “Uso de inteligência artificial generativa”, e a originalidade do conteúdo gerado foi assegurada pelos autores.

Título: Por favor, revise o título do trabalho para que ele reflita claramente o tema principal, o delineamento, o local e o ano da pesquisa.

Resultados: As inferências e interpretações apresentadas devem ser restritas à discussão entre a página 6, linha 23, e a página 7, linha 19.

Descrição dos desfechos: Verifique a descrição dos desfechos, especialmente os negativos. A linguagem deve ser precisa e sem ambiguidades. Certifique-se de revisar e descrever o desfecho do estudo de forma clara.

Citações: Evite mencionar organismos, autores e nomes de relatórios no texto. Por exemplo, na página 5, linha 27, ao mencionar a classificação referida por Scattolin, Diogo e Colombo (2007), essa informação deve constar apenas nas referências.

Formatação: Verifique a formatação de sinais matemáticos, garantindo que não haja espaços antes e depois, especialmente nos resultados, como em “p valor” e “n”, que atualmente apresentam espaços inadequados.

Redação: Evite o uso do termo “respectivamente”. Reformule as frases para apresentar os dados de forma mais próxima à sua correspondência, evitando confusões na leitura, como na página 7, linhas 26 e 27.

Artigos indefinidos: Revise e remova artigos indefinidos onde seu uso for inadequado, como em “uma prevalência”, “um aumento”, e “uma redução”. Exemplos a serem corrigidos estão na página 6, linha 30, e página 8, linha 33.

